# Application of Photo Texture Analysis and Weather Data in Assessment of Air Quality in Terms of Airborne PM_10_ and PM_2.5_ Particulate Matter

**DOI:** 10.3390/s21165483

**Published:** 2021-08-14

**Authors:** Monika Chuchro, Wojciech Sarlej, Marta Grzegorczyk, Karolina Nurzyńska

**Affiliations:** 1 Department of Geoinformatics and Applied Computer Science, Faculty of Geology, Geophysics and Environment Protection, AGH University of Science and Technology, al. Mickiewicza 30, 30-059 Krakow, Poland; wsarlej@agh.edu.pl (W.S.); marta.manka.95@gmail.com (M.G.); 2 Institute of Informatics, Faculty of Automatic Control, Electronics, and Computer Science, Silesian University of Technology, Akademicka 16, 44-100 Gliwice, Poland; Karolina.Nurzynska@polsl.pl

**Keywords:** classification, particulate matter, regression, texture analysis

## Abstract

The study was undertaken in Krakow, which is situated in Lesser Poland Voivodeship, where bad PM_10_ air-quality indicators occurred on more than 100 days in the years 2010–2019. Krakow has continuous air quality measurement in seven locations that are run by the Province Environmental Protection Inspectorate. The research aimed to create regression and classification models for PM_10_ and PM_2.5_ estimation based on sky photos and basic weather data. For this research, one short video with a resolution of 1920 × 1080 px was captured each day. From each film, only five frames were used, the information from which was averaged. Then, texture analysis was performed on each averaged photo frame. The results of the texture analysis were used in the regression and classification models. The regression models’ quality for the test datasets equals 0.85 and 0.73 for PM_10_ and 0.63 for PM_2.5_. The quality of each classification model differs (0.86 and 0.73 for PM_10_, and 0.80 for PM_2.5_). The obtained results show that the created classification models could be used in PM_10_ and PM_2.5_ air quality assessment. Moreover, the character of the obtained regression models indicates that their quality could be enhanced; thus, improved results could be obtained.

## 1. Introduction

Air quality in cities and suburban areas is a crucial and emerging problem for governments. Among various air pollutants, airborne particulate matter (PM) with diameters less than 10 micrometers (PM_10_) and less than 2.5 micrometers (PM_2.5_) are the most common pollutants in Polish cities. PM is a complex mixture of extremely small particles and liquid droplets made up of acids, organic chemicals, metal, soil, and dust particles. Sources of PM are both natural and anthropogenic. Man-made sources of PM include combustion in mechanical and industrial processes, vehicle emissions, and even tobacco smoke. Natural sources include volcanoes, fires, dust storms, and aerosolized sea salt [1].

PM has a considerable negative effect on human health, including increased rates of cardiovascular, cerebrovascular, and respiratory diseases [2,3]. Puentes et al. in [4] showed that every 50 µg/m^3^ increase in PM_10_ caused 4–12% growth of hospital visits for children with respiratory syndromes. Many techniques are available to measure the mass concentration of PM in air. The most popular methods include filter-based gravimetric methods [5], tapered element oscillating microbalance [6], beta attenuation monitoring [7], optical analysis [8,9,10], and black smoke measurement [11]. All these methods require sophisticated equipment, space to install, and staff to maintain the equipment and evaluate the data. A simple, fast, and cheap method of monitoring PM in the air has the potential to increase public awareness, alert those with respiratory diseases to take proper prevention measures, and provide local air quality data that are not otherwise available. Easy access to cheap cameras makes the described method easily accessible. The current methods, although undoubtedly more precise, are burdened with a very high cost of purchasing the equipment required for PM analysis. As indicated by the authors of the evaluation, the cost of purchasing a professional measuring device is so high that it becomes inaccessible for ordinary people [12].

The literature mentions the use of artificial neural networks, SVM, spatial interpolation models, and statistical models for air quality modeling. In neural network-based methods, Vahdatpour et al. proposed a method to estimate pollution forecast with Convolutional Neural Network based on sky images and Gabor transform [13,14]. Yang et al. presented shallow ResNet with layer enhancement for PM_2.5_ index prediction [15] based on image data from Beijing, Shanghai in China. The usage of MLP was presented by the authors in [16,17]. Their results demonstrated that the MLP approach obtains good PM levels prediction quality.

Liu et al. in [18] proposed PM_2.5_ level prediction with the SVR method based on six features (including textures) and weather data, time, and geographical location. The research was based on outdoor photographs from Beijing, Shanghai in China and Phoenix in US. In [19], the authors proposed a system to estimate air quality based on publicly available sky photos from Flickr and public webcams and statistics computed from sky pixels color values. The research of Sajjadi et al. in [20] presented PM_2.5_ and PM_10_ assessment in Sabzevar in Iran based on spatial interpolation models. Models of Radial Basis Functions, Inverse Distance Weighting, Ordinary Kriging, and Universal Kriging were used on 48 PM station data. The use of neural networks and public cameras or even smartphones with an integrated full-HD camera can provide an effective tool for assessing air quality [21,22]. Several forecasting models have been developed to assess levels of PM in atmospheric air without using photographs. In paper [23], the authors presentend generalized models with gamma distribution to predict daily average PM_10_ in Brno, Czech Republic. Models were created for daily averaged data from two stations using weather data and addictional seasonal informations. A similar approach was presented by the authors of [4]. The authors used a bivariate predictive model based on GBS distribution to predict next-day maximum PM_10_ and PM_2.5_ levels. In paper [24], the authors analyzed the impact of weather on PM level using generalized additive models. Finally, in [25], the authors presented a system to estimate PM_2.5_/haze level based on a single photograph. Experiments were performed both on synthetic and real datasets with depth trans and jcsb2014 methods.

The aim of the project was assessment of PM_10_ and PM_2.5_ pollution using image texture analysis and commonly available weather data (air temperature, precipitation, average wind speed). The authors aimed to verify whether it is possible to correctly forecast the pollution having information obtained from a simple camera, an image sensor that everyone has at home, and weather forecast one can obtain online. The task used the signal processing approach to derive discriminative features from images that are not visible with the naked eye. These data were combined with information collected by sensors built into commercial devices (Vaisala WXT520, Plantower PMS5003) that were measuring parameters such as wind, rain, pressure, temperature, and relative humidity. The assessment of air pollution with PM_10_ and PM_2.5_ was carried out quantitatively and qualitatively with the use of multilayer perceptron (MLP) artificial neural networks. Despite the fact that air quality in Krakow has improved in recent years, a tool that could quickly assess air quality anywhere and at any time of day would be useful. Such a tool could be based on regression or classification models that assess air quality based on texture and weather information. For example, the entire model could be integrated into a smartphone or tablet application.

## 2. Air Quality Assessment for the City of Krakow

Krakow agglomeration is located in the Lesser Poland Voivodeship; it is inhabited by around 770,000 people and covers an area of 327 km^2^. Air quality assessment for the city is based on the Regulation of the Minister of the Environment from 24 August 2012 on the topic of levels of certain substances in the air and EU directives for the protection of human health and plant protection [26,27,28,29] in which acceptable levels of atmospheric pollution are specified. The maximum yearly average concentration of PM_10_ equals 40 µg/m^3^, but the aforementioned regulation [28] also allows the daily average concentration to be exceeded (Table 1). The norm for PM_2.5_ concentration equals 25 µg/m^3^ without any exceptions.

Measurements of PM_10_ and PM_2.5_ content were carried out every day using continuous, high-quality, automatic or manual methods. The main factor affecting air quality in Lesser Poland Voivodeship is emissions from the municipal and household sectors. In the structure of pollutants, these emissions account for approximately 77% PM_10_, 88% PM_2.5_, 97% BaP, 14% NO_x_, and 65% SO_x_. An increased pollution level is especially visible in winter, when fuel consumption for heating increases due to low temperatures. In summer, emissions from the municipal and household sectors decrease; therefore, emissions only come from households that use solid-fuel furnaces to heat water. As in other highly polluted cities, such as Santiago, Chile, it should be noted that topographic (poor ventilation), meteorological (low temperature at winter), and socio-economic (coal-fired home heating) conditions negatively affect air quality in the city, especially during the winter period [4]. Another source of emissions that is visible especially in large cities and agglomerations is transport, which accounts for around 5% of PM_10_ emissions, 4% of PM_2.5_ emissions, and 44% of NO_x_ emissions for the whole voivodeship [30].

**Figure 1:**
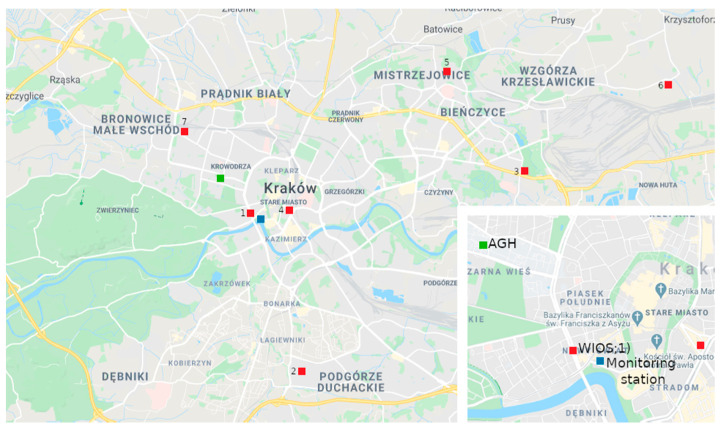
WIOS air quality station locations in Krakow.

**Figure 2:**
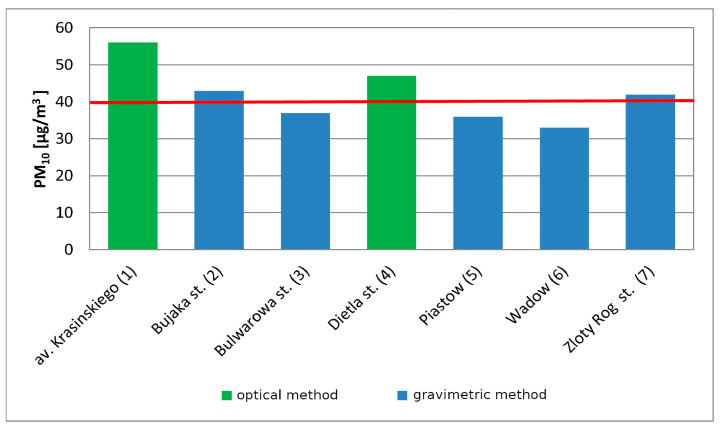
Concentration of PM_10_ at WIOS stations in Krakow in 2017.

To assess air quality in Krakow for PM_10_, measurement series from seven stations (two automatic optical and five manual gravimetrical) were collected and analyzed by the Province Environmental Protection Inspectorate. The locations of all stations are presented in Figure 1. The most frequently exceeded norm was observed at Krasinski Avenue [30], and the highest yearly PM_10_ number is also observed there (Figure 2).

To assess air quality in terms of PM_2.5_ particulate matter, measurement series from three stations (two automatic and one manual) were analyzed. The average annual concentrations ranged from 39 µg/m^3^ at Krasinski Avenue up to 27 µg/m^3^ at Bulwarowa Street in 2017 [30].

**Figure 3:**
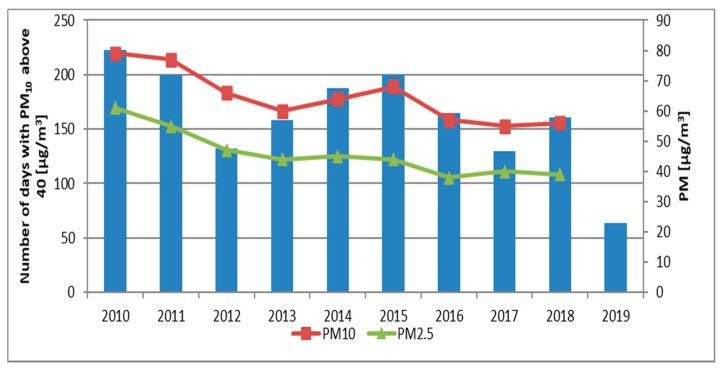
Days per year with exceeded norms of PM_10_ for Krakow agglomeration and PM_10_, PM_2.5_ concentrations at Krasinskiego.

The analysis of the number of exceedances of PM_10_ levels showed that the worst year in terms of air quality was 2010, which had 223 exceedances. The data show a decreasing trend, with a period of increased values in 2014–2015 (Figure 3) and a lower exceedance value in 2012. The concentrations of PM_10_ and PM_2.5_ show a clear decreasing trend (Figure 3), but a slight increase in concentrations was visible in the years 2014–2015.

## 3. Theory

In this paper, texture analysis using First-order Features, Gray-level Co-occurrence Matrix, and Grey Tone Difference Matrix methods was used to extract information from images. On the basis of the obtained results and weather data, regression and classification neural networks were created using multilayer perceptron. The task of the neural networks was to estimate PM_10_ and PM_2.5_ in atmospheric air and exceedances of air quality. In this section, the theory of used methds is presented.

### 3.1. Texture Analysis

The image processing literature illustrates multiple methods of describing the characteristics of a texture. The simplest method is image histogram analysis (e.g., First-order Features). More sophisticated approaches additionally analyze local changes in pixel intensities (e.g., Gray-level Co-occurrence Matrix); others try to mimic the way the human visual system works (e.g., Gray Tone Difference Matrix) [31,32]. Since the texture operator has already proved its sensitiveness for tiny changes resulting from noise introduction [33], it is believed the pollution recorded on images may also be visible. Moreover, in the presented research, a texture operator, which returns a short vector of values that could describe the image quality, was needed (which in assumption should vary depending on air condition). As a consequence, the authors have concentrated on early approaches of texture description and neglected the more up-to-date methods, e.g., Local Binary Patterns or Histogram of Oriented Gradients, that describe the image with extremely long feature vectors. Details of the methods chosen for this research are described in the following paragraphs.

The First-order Features method (FOF) is based on statistical information derived from a normalized image histogram. It denotes a gray-scale image whose resolution is *HxW*, and there are *G* pixel intensities. The normalized histogram is formulated as:

(1) $$p\left(i\right)=1/WH\;\sum_{x=1}^{W\;}\sum_{y=1}^{H\;}Ι\;\left(x,y\;\right)==i.$$

From such a histogram, the following features can be derived: mean, variance, kurtosis, skewness, energy, and entropy; their formulas are given in Equation (2) [34].



$$\mathrm{FOF}1:\mathrm{mean}:\;\;\;\;\mu =\overset{G-1}{\underset{i=0}{\sum }}ip\left(i\right)$$

$$\mathrm{FOF}2:\mathrm{variance}:\;\;\;\;\sigma^{2}=\overset{G-1}{\underset{i=0}{\sum }}\left(i-\mu^{2}\right)p\left(i\right)$$

$$\mathrm{FOF}3:\mathrm{kurtosis}:\;\;\;\;\mu_{3}=\sigma^{-3}\sum_{i=0}^{G-1}\left(i-\mu^{3}\right)p\left(i\right)b$$

$$\mathrm{FOF}4:\mathrm{skewness}:\;\;\;\;\mu_{4}=\sigma^{-4}\overset{G-1}{\underset{i=0}{\sum }}\left(i-\mu^{4}\right)p\left(i\right)-3$$

$$\mathrm{FOF}5:\mathrm{energy}:\;\;\;\;\;\;\;\;E=\overset{G-1}{\underset{i=0}{\sum }}{\left[p\left(i\right)\right]}^{2}$$

(2) $$\mathrm{FOF}6:\mathrm{entropy}:\;\;\;\;H=-\overset{G-1}{\underset{i=0}{\sum }}p\left(i\right)\log_{2}\left[p\left(i\right)\right]$$

In the Neighborhood Gray-tone Difference Matrix method (GTDM) [35], higher-order parameters are based on the histogram of differences between the intensity of the central pixel and its eight-sided neighborhood average. Such a definition mimics the way humans perceive brightness. From this data structure, five features are derived, which describe the general image quality: thickness, contrast, business value, complexity, and endurance. Equation (3) defines these features.

$$\mathrm{GTDM}1:\mathrm{coarseness}:\;\;\;\;{\left[\varepsilon +\overset{G_{h}}{\underset{i=0}{\sum }}p_{i}s\left(i\right)\right]}^{-1}$$

$$\mathrm{GTDM}2:\mathrm{contrast}:\;\;\;\;\left[\frac{1}{N_{g}\left(N_{g}\right)-1}\;\overset{G_{h}}{\underset{i=0}{\sum }}\overset{G_{h}}{\underset{j=0}{\sum }}p_{i}p_{j}{\left(i-j\right)}^{2}\right]\left[\frac{1}{n^{2}}\overset{G_{h}}{\underset{i=0}{\sum }}s\left(i\right)\right]$$

$$\mathrm{GTDM}3:\mathrm{business}\;\mathrm{value}:\;\;\;\;\frac{\left[\sum_{i=0}^{G_{h}}p_{i}s\left(i\right)\right]}{\sum_{i=0}^{G_{h}}\sum_{j=0}^{G_{h}}i\;p_{i}-jp_{j}}$$

$$\mathrm{GTDM}4:\mathrm{complexity}:\;\;\;\;\left[\frac{\sum_{i=0}^{G_{h}}\sum_{j=0}^{G_{h}}\left(\mathrm{mod}\left(i,j\right)\right)}{n^{2}(p_{i}+p_{j)}}\left(p_{i}s\left(i\right)+p_{j}s\left(j\right)\right)\right]$$

(3) $$\mathrm{GTDM}5:\mathrm{strength}:\;\;\;\;\sum_{i=0}^{G_{h}}\sum_{j=0}^{G_{h}}(p_{i}+p_{j})\frac{{\left(i-j\right)}^{2}}{\varepsilon -\sum_{i=0}^{G_{h}}s\left(i\right)}$$

where

$p_{i}—\mathrm{the}\;\mathrm{probability}\;\mathrm{of}\;\mathrm{the}\;\mathrm{dependence}\;\mathrm{of}\;a\;\mathrm{pixel}\;\mathrm{on}\;\mathrm{intensity}\;i;$
s(i)—NGTDM intensity value calculated as ∑|i−Ai|;
Ai—the average intensity of the surrounding pixels without considering the center voxel (calculated from the intensity i).

The Gray-Level Co-occurrence Matrix (COM) is the third texture method used in this paper. This method was introduced by Haralick [36] and is based on a symmetrical co-occurrence matrix. Each matrix entry stores information about the number of occurrences of two-pixel intensities that index this element in an image. This definition makes it possible to memorize not only an image’s intensity distribution but also its spatial relation to the image. The matrix dimensions are related to the number of gray-level pixel values, *G*, and may be reduced to a smaller number to optimize the calculation time. In the presented research, *G* equals 64. To assure the rotational invariance of the method, Haralick et al. suggested incorporating into the co-occurrence matrix the information that is obtained from analyzing the pixel adjacency in four directions [36]. Moreover, it is possible to define the distance between pixels, which are assumed to be adjacent. In the presented experiments, this distance equals one. These authors also suggested deriving 14 features that describe various qualities of a texture. The detailed formulation is presented in Equation (4).

$$\mathrm{COM}1:\mathrm{Angular}\;\mathrm{Second}\;\mathrm{Moment}:\;\;\;\;\sum_{i}\sum_{j}p{\left(i,j\right)}^{2}$$

$$\mathrm{COM}2:\mathrm{Contrast}:\;\;\;\;\overset{N_{p}-1}{\underset{n=0}{\sum }}n^{2}\left\{\overset{N_{p}}{\underset{i=1}{\sum }}\overset{N_{p}}{\underset{j=1}{\sum }}p\left(i,j\right)\right\},\;\mathrm{mod}\left(i-j\right)=n$$

$$\mathrm{COM}3:\mathrm{Correlation}:\;\;\;\;\frac{\sum_{i}\sum_{j}\left(ij\right)p\left(i,j\right)-\mu_{x}\mu_{y}}{\sigma_{x}\sigma_{y}}$$

$$\mathrm{COM}4:\mathrm{Sum}\;\mathrm{of}\;\mathrm{Squares}:\mathrm{Variance}:\;\;\;\;\sum_{i}\sum_{j}{\left(i-\mu \right)}^{2}p\left(i,j\right)$$

$$\mathrm{COM}5:\mathrm{Inverse}\;\mathrm{Difference}\;\mathrm{Moment}:\;\;\;\;\sum_{i}\sum_{j}\frac{1}{1+{\left(i-j\right)}^{2}}p\left(i,j\right)$$

$$\mathrm{COM}6:\mathrm{Total}\;\mathrm{Average}:\;\;\;\;\overset{2N_{p}}{\underset{i=2}{\sum }}ip_{x+y}\left(i\right)$$

$$\mathrm{COM}7:\mathrm{Sum}\;\mathrm{of}\;\mathrm{Variance}:\;\;\;\;\overset{2N_{p}}{\underset{i=2}{\sum }}{\left(i-f_{s}\right)}^{2}\;\;p_{x+y}\left(i\right)$$

$$\mathrm{COM}8:\mathrm{Sum}\;\mathrm{of}\;\mathrm{Entropy}:\;\;\;\;-\overset{2N_{p}}{\underset{i=2}{\sum }}\;p_{x+y}\left(i\right)\log\left\{p_{x+y}\left(i\right)\right\}$$

$$\mathrm{COM}9:\mathrm{Entropy}:\;\;\;\;-\underset{i}{\sum }\underset{j}{\sum }p\left(i,j\right)\log\left(p\left(i,j\right)\right)$$

$$\mathrm{COM}10:\mathrm{Difference}\;\mathrm{Variance}:\;\;\;\;\overset{N_{p}-1}{\underset{i=0}{\sum }}i^{2}p_{x+y}\left(i\right)$$

$$\mathrm{COM}11:\mathrm{Difference}\;\mathrm{Entropy}:\;\;\;\;-\overset{N_{p-1}}{\underset{i=0}{\sum }}i^{2}\;\;p_{x-y}\left(i\right)\log\left\{p_{x-y}\left(i\right)\right\}$$

$$\mathrm{COM}12:\mathrm{Information}\;\mathrm{Measures}\;\mathrm{of}\;\mathrm{Correlation}\;1:\;\;\;\;\frac{HXY-HXY1}{\max\left\{HX,HY\right\}}$$

COM13:Information Measures of Correlation 2:    (1−exp[−2(HXY2−HXY)])12

COM14:Max. Correlation Coefficient:    (second biggest value of Q)12

$$HXY=-\underset{i}{\sum }\underset{j}{\sum }p\left(i,j\right)\log\left(p\left(i,j\right)\right)\;HX,\;HY—\mathrm{entropies}\;p_{x},\;p_{y}$$

$$HXY1=-\underset{i}{\sum }\underset{j}{\sum }p\left(i,j\right)\log\left(p_{x}\left(i)\;p_{y}(j\right)\right)$$

$$HXY2=-\underset{i}{\sum }\underset{j}{\sum }p_{x}\left(i)\;p_{y}(j\right)\log\left(p_{x}\left(i)\;p_{y}(j\right)\right)$$

(4) $$Q\left(i,j\right)=\sum_{k}\frac{P\left(i,k\right)P\left(j,k\right)}{P_{x}\left(i\right)P_{y}\left(k\right)}.$$

where

μ—average value, σ—standard deviation;
$\;p_{x}—\mathrm{partial}\;\mathrm{probability}\;\mathrm{density}\;\mathrm{function};$
x, y—input coordinates in the co-occurrence matrix;
$p_{x+y}\left(i\right)—\mathrm{probability}\;\mathrm{of}\;\mathrm{the}\;\mathrm{sum}\;\mathrm{of}\;\mathrm{the}\;x\;\mathrm{and}\;y\;\mathrm{coordinates}\;\mathrm{from}\;\mathrm{the}\;\mathrm{matrix}\;\mathrm{of}\;\mathrm{co}-\mathrm{occurrences};$

### 3.2. Correlation

The correlation coefficient describes the statistical relationship between features. Pearson’s correlation is used to measure the statistical relationship or association between two continuous variables. It provides information about the magnitude of the association and the direction of the relationship [37]. Calculation of Pearson’s correlation coefficient requires the assumption that two samples are normally distributed. If normality is violated, Pearson’s correlation coefficient gives unreliable results. Hence, an alternative to Pearson’s correlation coefficient may be Spearman’s rank correlation. The dependence of ordinal variables is designated as rank correlation, and their intensity is represented by correlation coefficients [37]. Correlations with *p* ≤ 0.05 are considered statistically significant, which corresponds to correlation values greater than 0.195.

### 3.3. Artificial Neural Networks

Artificial neural networks are a powerful data modeling tool with a high proven efficiency in dealing with nonlinearity in a dataset as well as complex problems in the classification, regression, and clustering fields [17,37,38]. An extensive description of neural networks has been provided by the authors in [15,17,37,38,39]. For the regression problem, a neural network with a multilayer perceptron (MLP) was selected [16,17,40]. Network training was performed with the Broyden–Fletcher–Goldfarb–Shanno (BFGS) algorithm, which is an iterative method for solving unconstrained nonlinear optimization problems. The BFGS is a memory-efficient training algorithm usually used for nonlinear least squares and may require in a smaller number of iterations to train a neural network [38]. The sum of squares (SOS) was selected as the error function. Initially, the number of neurons in the input layer depended on statistically significant correlations: one neuron for each quantitative variable. The optimal number of variables used in the model was based on sensitivity analysis, so the number of neurons in the input layer was changeable. Variables that did not improve the quality of the obtained networks were removed from the neural network models. The constructed neural networks included one layer of hidden neurons with five to 25 neurons and one neuron in the output layer [17,38].

Originally, two types of neural networks were considered for the classification problem: a multilayer perceptron and a network with a radial basis function (RBF) [39]. After preliminary analyses, it turned out that the quality of the RBF network was lower than that of MLP; therefore, the multilayer perceptron was chosen. Moreover, for neural networks classification, sensitivity analysis was used to select the optimal number of variables, and the number of neurons in the hidden layer was from five to 25 neurons. The output layer contained as many neurons as the air quality classes (for PM_10_—3, for PM_2.5_—2).

The quality of the regression neural networks was expressed as the value of Pearson’s correlation coefficient between the data and the model, and the Coefficient of Determination (R^2^). Mean Error (ME), and Mean Absolute Percentage Error (MAPE) were used additionally for test datasets. The quality of the classification networks was expressed as the percentage of correct classifications, which corresponds with accuracy * 100%. Additionally, Area Under Curve (AUC) was used to assess the ability of a classifier to distinguish between classes.

## 4. Proposed Method

**Figure 4:**
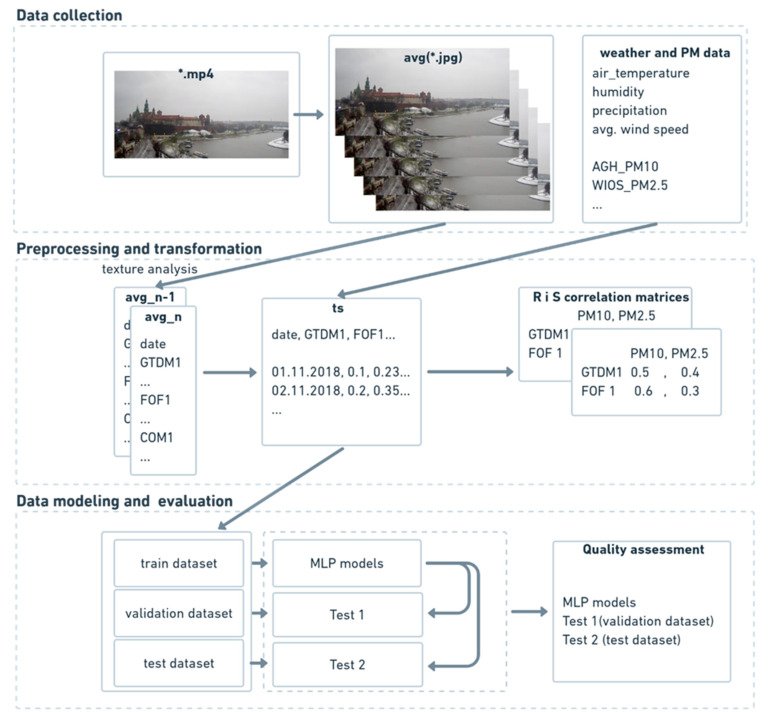
Block diagram of the proposed method.

For this project dataset of images, numerical datasets of weather data and air pollution with PM_10_ and PM_2.5_ data have been used. Proposed method for estimating PM_10_ and PM_2.5_ in atmospheric air and exceedances of air quality was based on the Cross-Industry Standard Process for Data Mining (CRISP-dm) methodology. Figure 4 illustrates the block diagram of the proposed method. The authors described the phases presented in Figure 4 in the following parts of this paper. The proposed method is a transitive method between the use of neural networks to predict air quality based on photos presented in publications [13,14,15] and the approach based on modeling numerical data presented by the authors in [16,23,24]. In the first step, texture analysis for each of the average photo frames has been performed, using three complementary methods. Thanks to the selected methods, a single value for each texture feature was obtained. The next step was to combine the results from the texture analysis, weather, and air pollution data. Then, regression and classification MLP neural networks were performed for numerical data. The selected method allowed for the creation and preliminary evaluation of a single neural network in less than 1 s. In the last step, the quality of selected neural network models was assessed.

### 4.1. Data Collection

**Figure 5:**
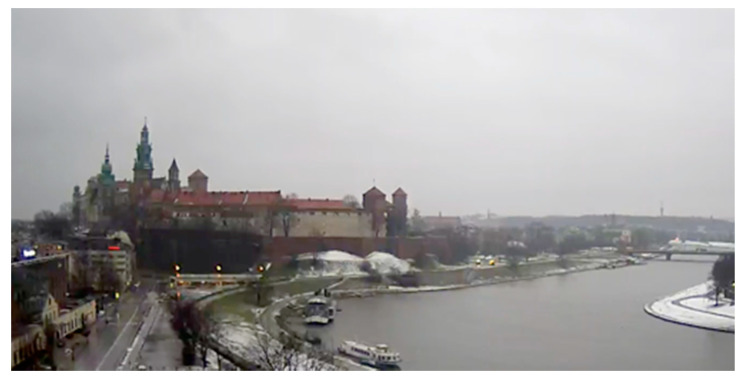
Single frame used in visual inspection of air quality.

Data analysis was performed on good-quality image data that were acquired daily. Image data acquisition was performed periodically for 100 days (from 21 November 2018 to 28 February 2019) at sunrise (between 7 and 7.30 AM, UTC+1) from the “Wawel castle and the Vistula bend” city monitoring station, whose coordinates are 50°03′ N 19°55′ [41]. The automated process was downloading videos from the camera according to the aforementioned schedule. Short *.mp4 files (movies with resolution 1920 × 1080 px) were saved in the disk space, from which five random frames were extracted and averaged. Each frame contains the same view presented in Figure 5. Images carry unstructured sets of pixels; however, each image has a texture that has been analyzed in further steps. On the other hand, weather parameters collected by sensors have been provided as structured *.csv files. The combination of both sources done by the automated process gave the results presented below. To ensure the best possible convergence of parameters, weather and air quality data were collected from two weather stations located in the neighborhood of the image acquisition location, which is presented in Figure 1.

Weather data were obtained from the weather station run by the Environmental Physics Group of AGH University of Science and Technology in Krakow (AGH), which is shown on the map (Figure 1) as a green square [42]. The averaged results of parameter measurements from a full hour were registered. For this period (21 November 2018 to 28 February 2019), 7:00 a.m. was established as a measuring point. The location of the station is 50°04′ N 19°55′ E, and its foundation height is 220 m a.s.l. The weather station provided meteorological information, including average air temperature, average relative humidity, average atmospheric pressure, average wind speed, maximum speed of wind gusts, precipitation and air quality status assessed using the PM_10_ indicator. Weather and airborne particulate matter measurements were provided by a Vaisala WXT520 automatic weather station and a Plantower PMS5003 [42].

Additional air quality status information was obtained from the station of the Voivodeship Inspectorate of Environmental Protection in Krakow (WIOS), located at Krasinski Avenue (Figure 1) [43]. The measuring point coordinates are 50°03′ N 19°55′ E; its foundation height is 207 m a.s.l. The station is located close to the “Wawel Castle and Vistula bend” monitoring point, which provided images from webcams. The measuring station at Krasinski Avenue recorded the following parameters: nitrogen oxide content, carbon monoxide content, nitrogen dioxide content, benzene content, PM_10_, and PM_2.5_ indicators.

### 4.2. Preprocessing

Every object has a texture that can be used to characterize it. Even if one considers an object to have no texture, image processing methods consider it to have a plain texture. An example of a plain texture is a photograph of a clear sky on a sunny day. When clouds are visible, each of them may have a different texture. Changes in air pollution are also noticeable in photographs and can be reflected as changes in texture. The project used three complementary texture methods presented in Section 3 on average images from the camera. The textures features were saved into structured *.csv file. In the next step, files with weather data, texture features, and PM data were combined into one file with a time index (see Figure 4). Such a prepared file was used in correlation analysis and modeling.

### 4.3. Modeling and Evaluation

Regression and classification of the neural network models were used to assess the possibility of predicting the content of PM_10_ and PM_2.5_ in atmospheric air. Moreover, based on image texture parameters and basic meteorological data (air temperature, average wind speed, humidity, precipitation), they were also used to predict the exceedances of air quality standards.

The analyzed particulate matter data from each of the measuring stations were divided into 3 subsets. Since the analyzed data are a time series, the data were not randomly divided into subsets. The oldest 70% of the observations were assigned to the training set, another 15% was assigned to the validation set, and the newest observations were assigned to the test set. One thousand different regression neural networks with different architectures were made for each parameter. The neural network was created using a training dataset with the automatic Data Mining toolbox in Statistica 13.1. The created neural networks had a three-layer structure with one hidden layer. In the hidden layer, we tested from 5 to 25 neurons. The maximum number of epochs was 200, and the stop criterion was 0.00001. The initial weights were random using normally distributed values within a range whose mean is zero and standard deviation is equal to one. These networks differed also in the activation functions of neurons in the hidden layer and the output layer. Each neural network was created based on a training dataset. Successively, the fitted model is used to predict the responses for the observations in a second dataset called the validation dataset. The validation dataset provides an unbiased evaluation of a model fit on the training dataset. In the last step, an unbiased evaluation of a final model fit on the training dataset is performed with the test dataset.

Of all the created networks, the one with the highest quality values for all three datasets and the lowest number of neurons in the hidden layer was selected for each predicted PM dataset, to present in this paper.

Classification methods are sensitive to the unevenness of the occurrence of classes in subsets. Therefore, for the classification methods, only a random selection of observations for individual sets was used to better balance the observations belonging to individual classes. For the classification models, the quantitative data were changed to 3 PM_10_ classes. The good air quality class was defined as PM_10_ lower than 50 µg/m^3^; the poor air quality class was PM_10_ between 50 and 100 µg/m^3^; and the very poor-quality class was PM_10_ higher than 100 µg/m^3^. This division was made based on the ordinance of the Polish Minister of the Environment [28,29]. For suspended particle matter with a diameter smaller than 2.5 micrometers, two classes were distinguished: air quality is considered good if dust content is lower than 25 µg/m^3^, and it is considered poor for higher values [28]. The same as for the regression models, one thousand models were created automatically for each PM dataset, with the same conditions as presented in previous paragraph; from these, only the ones with the highest quality were used in the tests.

### 4.4. Software

The project was implemented on computers with a Windows 10 × 64-bit system with an Intel Core i7-3630QM CPU 2.4 GHz processor, 16 GB RAM, and a Windows 10 × 64-bit system with an Intel Core i7-10710U CPU 1.10 GHz processor, 16 GHz RAM. In this project, we used specialized software, computing platforms, and programming languages for statistical computing and graphics. For texture analysis, we used MATLAB 2016a, which is a programming and numeric computing platform. Correlation matrices and quality assessment were prepared in the R × 64 4.0.2 language with function cor from stats package version 3.6.2, and caret package version 6.0–88. Neural networks were created in the Statistica 13.1 64-bit program with SANN toolbox for automatic neural network building.

## 5. Results

Data analysis was performed to assess the relationship between dust suspended in atmospheric air and the texture parameters calculated from the averaged film frames recorded at sunrise and the basic weather data: air temperature, humidity, precipitation, average wind speed. The following analyses were carried out in sequence: Pearson’s linear correlation, Spearman’s rank nonlinear correlation, MLP regression neural network model, and MLP neural network classification models.

**Figure 6:**
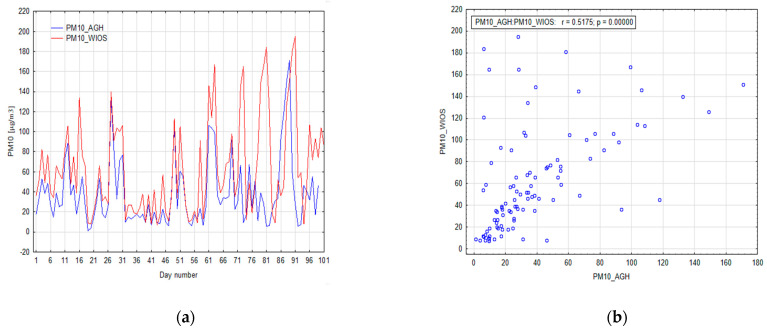
Correlation between AGH and WIOS stations for PM10: (**a**) time series comparison, (**b**) correlation plot.

In the first step, the relationship between the measurement stations for the recorded suspended PM_10_ dust measurements was determined. The linear correlation coefficient between PM_10_ at AGH station and PM_10_ at WIOS is 0.52. The value of the Spearman’s rank correlation is slightly higher, which indicates the presence of nonlinear relationships between these stations (0.6). This is a relatively low similarity when it is considered that the stations are only about one kilometer apart. The analysis of the line graph of both PM_10_ datasets shows that the data are positively correlated with each other (Figure 6a), but there are also significant differences in mean values and variation between those two stations (Figure 6a). Differences between measurements are smaller for low PM_10_ values (Figure 6b). As the measured values increase, the difference between the PM_10_ datasets also increases. In Figure 6a, two periods are visible with very high differences between the PM_10_ WIOS station and PM_10_ AGH station values. The first period is 70–73 observations with a maximum of 73 observations. The difference between measurements is 155.6 µg/m^3^. The second period is 76–81 observations with a maximum of 81 observations. The difference between measurements is 178 µg/m^3^. Both days with very high differences in measurements are days with negative air temperature lower than −5 °C, with an average wind speed lower than 1.5 m/s and without precipitation. Both periods are in a validation dataset. This may be related to the fact that the WIOS station is located in a green area in the middle of a busy road, while the AGH station is located near a less busy road on the AGH campus.

Based on the correlation analysis, we can conclude that there is a significant variation in PM content even over short distances; therefore, the models will be local in nature and will only be accurate in the immediate vicinity. In the next step, the relationship between the measurements for the WIOS station was computed. The Pearson’s correlation coefficient between PM_2.5_ and PM_10_ is 0.89. The value of the Spearman’s rank correlation is slightly lower, which indicates the presence of strong linear relationships between these stations (0.87).

The correlations between PM_10_, PM_2.5_, and texture parameters as well as air temperature, precipitation, and air humidity were calculated. Pearson’s linear correlation coefficient was used to assess linear relationships; Spearman’s rank coefficient was used to assess nonlinear relationships. In the Pearson’s correlation coefficients, the results with the highest correlation coefficient values for texture parameters (Table 2) were obtained using the GTDM method. All texture parameters are statistically significant with the PM_10_ and PM_2.5_ datasets. The highest correlation coefficient values were observed for PM_10_ from the WIOS station. In the FOF method, only two parameters correlated at a significant level for PM_10_ from the WIOS station (FOF1, FOF3). PM_10_ from the AGH station and PM_2.5_ were not significantly correlated with First-order Feature texture data. In Haralick’s method, almost all texture parameters correlated with PM_10_ WIOS; only correlations with COM1, COM8, and COM14 were not statistically significant (*p* > 0.05). Pearson’s correlation coefficients obtained for PM_2.5_ at WIOS and PM_10_ at AGH were weaker: 4% and 10%, respectively. Furthermore, the number of statistically significant correlations decreased: 20 for PM_10_ WIOS, 15 for PM_2.5_ WIOS, and 12 for PM_10_ AGH (Table 2). The highest Pearson’s correlation coefficient value was observed for the correlation between average wind speed and PM_10_ at WIOS station (−0.626). Moreover, Pearson’s correlation coefficient between PM_2.5_ from WIOS station and the average wind speed equals −0.58. It is visible that a medium-strength negative relationship exists between PM data and average wind speed. The highest correlation between PM datasets and texture parameters was observed between PM_2.5_ from WIOS station and GTDM2 (−0.485).

When comparing the results of the two correlation methods, the Spearman correlation coefficient was found to have higher values. The number of statistically significant correlations increases for PM_10_ from the AGH station. For all stations, the FOF4 and COM1 Spearman rank correlation coefficients are higher than Pearson’s correlation coefficients and are statistically significant.

For PM_10_ AGH, four more statistically significant correlations were measured with Spearman rank correlation than with the Pearson correlation coefficient: FOF4, COM1, COM3, and COM13. The highest changes in correlation values between the Pearson and Spearman correlation coefficients are visible for Haralick’s texture parameters: 0.241 (correlation was measured between COM10 and PM_10_ WIOS). When comparing the results of the two correlation methods, higher values for Spearman’s correlation coefficient are visible. The number of statistically significant correlations increases especially for PM_10_ from AGH station. For all stations, the FOF4 and COM1 correlation values increased and became statistically significant compared to the Pearson correlation coefficient. For PM_10_ from AGH station, four more statistically significant correlations were measured with Spearman rank coefficient (FOF4, COM1, COM3, COM13).

Due to the nonlinear relationships between PM data and texture parameters, a multilayer perceptron was used as a regression model. Texture parameters and weather data were chosen as independent variables, and they showed a statistically significant correlation with PM data. The number of neurons in the input layer equals the number of variables in the model. The variables used in the models are marked in dark blue italics in Table 2. From the 1000 neural networks that were created for each PM dataset, only the one with highest quality was chosen for tests.

The regression neural network prediction for PM_10_ values from the AGH measuring station consisted of 17 neurons in the input layer. The best network presented in the results had one hidden layer consisting of seven neurons, and an output layer with one neuron. An exponential function was chosen as the hidden layer activation function, and the neuron activation function in the output layer was linear. Learning, validation and testing qualities are high: 0.9, 0.8, and 0.85, respectively. The neural network algorithm required 57 epochs. The neural network that predicted PM_10_ values from WIOS station had two more neurons in the input layer and two fewer neurons in the hidden layer. The activation functions are the same as for the first neural network. Learning quality is similar to the first neural network (0.89); validation quality is higher (0.9); however, testing quality is lower (0.73). This neural network required 34 epochs. The last neural network, which predicted PM_2.5_ values, had 14 neurons in the input and hidden layers, and one neuron in the output layer. Its activation functions differ from the previous networks: in the hidden layer, we used the logistic function; in the output layer, we used the hyperbolic tangent; the learning process takes 26 epochs. The learning, validation, and testing qualities are the lowest from all the three neural networks: 0.8, 0.84, and 0.63, respectively. The coefficient of determination (R^2^) was computed for learning, validation, and test datasets. For PM_10_, AGH equals 0.81, 0.64, and 0.72, respectively. The R^2^ for PM_10_ WIOS are 0.79, 0.81, and 0.53. Lower R^2^ values were computed for PM_2.5_ WIOS: 0.64, 0.70, and 0.40.

**Figure 7:**
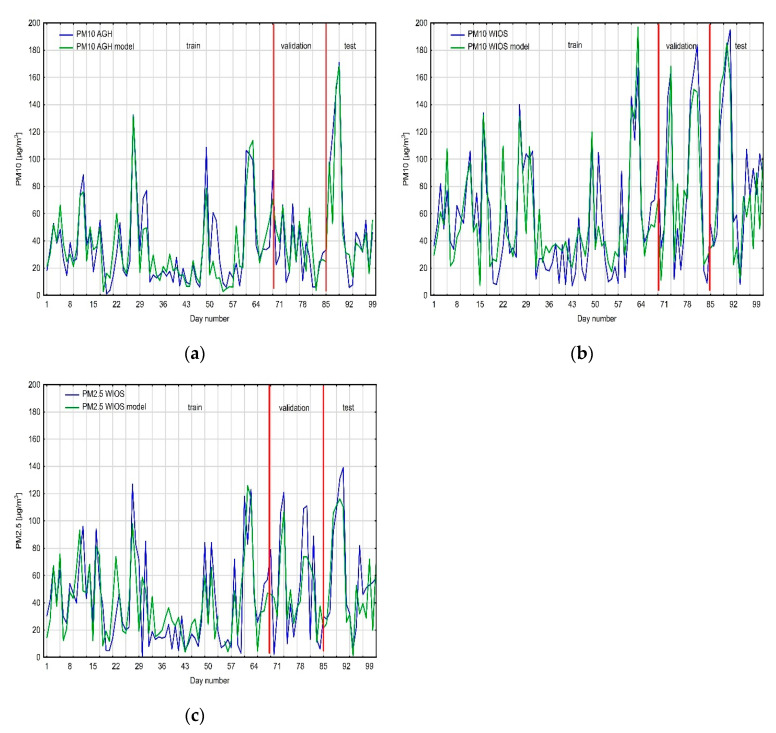
Comparison of the obtained models against real data: (**a**) PM_10_ AGH, (**b**) PM_10_ WIOS, and (**c**) PM_2.5_ WIOS.

Moreover, the models’ qualities were checked with mean error (ME) in the test dataset and mean absolute percentage error (MAPE). The MAPE values for the test dataset equal 27% (PM_10_ AGH), 13% (PM_10_ WIOS), and 19% (PM_2.5_ WIOS). The MEs for the test datasets equal 3.7, 5.5, and 3.9, respectively. The high ex-post measure values for the created models relate to differences in the dataset. The test datasets had higher variability than the training datasets for all PM data (Figure 7).

Air quality assessment of PM_10_ and PM_2.5_ exceedances was performed based on the MLP network. According to the ordinance of the Minister of the Environment, the PM data were divided into three classes (PM_10_) and two classes (PM_2.5_). The number of classes determined the number of neurons in the created networks’ output layers.

The best three neural networks were tested. The architecture differences between these neural networks are significant. The PM_10_ AGH neural network has 17 neurons in the input layer and nine neurons in the hidden layer. All statistically significantly correlated variables were used in the model (Table 2). The activating functions of the neurons were hyperbolic tangent and softmax. A more extensive network was obtained for PM_10_ WIOS, in which the input layer had one more neuron and the same number of neurons in the hidden layer as PM_10_ AGH. This network is additionally distinguished by the functions of the activating neurons in the hidden and output layers: logistics and hyperbolic tangent, respectively. The last PM_2.5_ WIOS neural network had 17 neurons in the input layer and 12 neurons in the hidden layer, and the activation functions were hyperbolic tangent and softmax, respectively.

The obtained percentage of correct classifications (accuracy * 100%) in the learning datasets varies from 87.3% for PM_10_ AGH to 94.4% for PM_2.5_ WIOS. For PM_10_ WIOS, the percentage of correct classification is 92%. The percentage of misclassifications is evenly distributed over the classes. The percentage of correct classifications in the validation datasets are 92% for PM_10_ AGH, 71% for PM_10_ WIOS, and 85% for PM_2.5_ WIOS. The quality of the neural networks in the test dataset is lower than in the learning dataset, but it is still high, and in the worst case (PM_2.5_ WIOS), it achieves 80% of correct classifications. The best results were obtained by the network that predicts PM_10_ AGH class. The test set achieved 92.9% correct classifications. PM_10_ WIOS obtained 85.7% correct classifications in the test dataset. The Area under Curve (AUC) values computed for whole datasets are 0.792 for PM_10_ AGH, 0.802 for PM_10_ WIOS, and 0.928 for PM_2.5_ WIOS.

The created confusion matrix for the test dataset shows that no error repeats constantly (Table 3). All the neural networks have one wrong classification, where poor air quality was classified as good. Two observations classified as “good” were classified as poor in PM_2.5_ WIOS, thus giving a total of only 80% of correct classifications. In addition, for PM_10_ WIOS, one additional wrong classification was observed: poor air quality was classified as very poor (Table 3).

## 6. Conclusions, Limitations, and Future Research

The issue of air pollution, including the particulate matter, has been extensively presented in the literature [1,2,3,24]. This is a key issue for most urbanized areas, especially for big agglomerations [4,14,16,23,24,44]. Air pollution in Poland is a big problem, especially in urbanized areas [22]. In the analyzed period in the city of Krakow, exceedances of the permissible standards presented in Table 1 were observed over 100 times a year (Figure 3). Due to frequency with which air quality limits were exceeded, cheap and effective tools are needed to quantify and qualify air quality.

The assessment of PM air pollution is commonly carried out using various types of methods discussed in the literature [5,6,7,8,9]. Carretero-Peña et al. (2019) and Sarimveis et al. (2006) proposed assessing air quality using image analysis. The assessment of air pollution with particulate matter is often based on images and images analysis. Papers presented models based on images analysis with Gabor filter [13], conversion to gray scale, and the Otsu method [18]. Liu et al. also proposed using six image features: transmission, whole image and local image contrast, entropy, sky smoothness, and color. The authors in [25] used Dark Channel Prior for transmission matrix estimation from multiple scene images. In paper [45], the authors used the Gray-level Co-occurrence Matrix method (COM) for texture feature extraction in PM assessment. In this paper, we used COM and two additional complementary textures methods: FOF and GTDM. Additionally, in previous research [20], RBF neural networks were used for the quantitative estimation of air quality, which was assessed with quality measures of ex-post forecast assessment. This paper also proposes the use of artificial neural networks; however, in the work of Ordieres et al., the MLP multilayer perceptron proved to be a better solution [17]. The use of MLP neural networks to predict air pollution on the basis of numerical data was presented by the authors in [16,17]. In paper [16], the authors use numerical variables and a K-mean algorithm for PM_10_ prediction with MLP and MLR. The R was between 0.67 and 0.77, depending on measurement localization for the regression model. Better regression results were obtained with CNN models with the PMIE method: R^2^ was between 0.68 (multiple-scene images) and 0.91 (single-scene images) for PM_2.5_ prediction [15]. The main limitation in using CNN is a small dataset of images. In paper [13], the authors presented shallow (Random Forest) and deep classifiers (CNN) for five-class air quality assessment for an Air Quality Index (AQI). The authors used a method for multiple-scene images and obtained an AUC of 0.6 and an accuracy of 0.53. A very similar approach to modeling was used in this article by adding texture features as additional variables. Additionally, a similar application of texture analysis in PM_2.5_ prediction was presented in [18] for images from China and United States. The main difference between [18] and this paper is in the used methods: six texture features versus three texture methods. Quality SVR models for Beijing were between 0.68 and 0.7 R^2^, for Shanghai, between 0.72 and 0.76 R^2^. The air quality analysis was based on analysis of textures on averaged images with full HD resolution: 1920 × 1080 px. The texture features were measured using three methods: The First-order Features, the Neighborhoods Gray-tone Difference Matrix method, and the Gray-Level Co-occurrence Matrix. For each of the three methods, statistically significant nonlinear correlations were demonstrated between the texture parameters and PM_10_ AGH, PM_10_ WIOS, and PM_2.5_ WIOS data. The correlations are weak to moderate. The highest correlation value is −0.518 between COM2 and PM_10_ WIOS. Based on the results obtained from the correlation analysis, two types of neural networks were created: regression and classification. Both types of networks had a satisfactory quality. The regression models obtained quality (R) in the 0.63–0.85 range for the test sets. However, the R^2^ vary from only 0.4 for PM_10_ AGH in the test dataset to 0.81 for PM_10_ AGH in the learning dataset, and PM_10_ WIOS in the validation dataset. Relatively low R^2^ values for PM_10_ AGH show model weakness, especially in validation and test datasets. What is more, the calculated MAPE value exceeds 10% for all the regression networks, which indicates that they will probably not be good enough to use, despite the relatively low average errors of these networks: PM_10_ AGH—3.7; PM_10_ WIOS—5.5; PM_2.5_ WIOS—3.9. The datasets used in the tests have high variation and higher mean values than in the learning datasets, so the neural network algorithms did not correctly predict data variation. This could be changed by using longer time series in each dataset. Better results were obtained with the classification models than with the regression models. The worst quality was obtained for the PM_2.5_ WIOS classification in the test dataset: 80% (Table 3). The qualities of other neural networks are equal and higher than 85%. The higher the AUC, the better the performance of the model at distinguishing between classes. The lowest AUC was calculated for PM_10_ AGH (0.792). The highest AUC equals 0.928 and was calculated for PM_2.5_ WIOS. High AUC values indicate a good fit of the classification models to the data.

The results of this study indicate that photo texture analysis could be useful in air quality assessment. All chosen texture methods were useful in the performed analysis. Additionally, it is possible to predict air quality exceedances by analyzing textures in HD photos with basic weather data as additional information. It is also possible to predict the values of PM_10_ and PM_2.5_ in atmospheric air, but these results have a greater error. This error could probably be minimized by using a longer period of data in the learning dataset that contains all seasons, or higher resolution photos, e.g., 4 K.

The obtained models could be used as part of an application for air quality control using smartphone camera photographs, especially for checking air quality because of the high quality and sensitivity of the PM_10_ model (higher than 90%). Currently, image sensors are widely accessible, for example, in online cameras and smartphones [21]. Existing infrastructure could be used to provide more photos to create better air quality models. Classification models could be even implemented in mobile phone applications, which will make analysis more accessible for end users. Future work should focus on building a process that will continuously examine images captured at different locations. Such a constant stream of input data combined with weather data will give a much better model that will assess air quality over a larger area. The limitation of this approach is not only the quality of the camera sensor but also the registered image itself. Night-time photos are not providing enough information for the model; the same applies to photos taken in bad weather conditions. Both of these aspects can be eliminated with the use of appropriate lighting and background, but at the same time, it would make this method much more difficult and more expensive to implement.

## Figures and Tables

**Table 1:** Acceptable daily and annual maximum levels of PM_10_ concentration in the air.

Average Period of Concentration	Permissible Level of PM_10_ in the Air [µg/m^3^]	Allowed Frequency of Exceeding the Permissible Level of PM_10_
24 h	50	35 times
Calendar year	40	N/A

**Table 2:** Pearson’s (R) and Spearman (S) correlation coefficients between PM_10_ and PM_2.5_ texture parameters and weather data. Statistically significant correlations with *p* ≤ 0.005 are marked in bold.

Parameter	Cor.	PM_10__AGH	PM_10__WIOS	PM_2.5__WIOS
Average hourly air temperature	R	**−0.224**	**−0.300**	**−0.284**
	S	*−0.270*	*−0.294*	**−0.238**
Average hourly wind speed	R	**−0.487**	**−0.626**	**−0.580**
	S	*−0.526*	*−0.628*	*−0.550*
Average hourly relative humidity	R	−0.016	−0.112	−0.047
	S	0.004	−0.169	−0.068
GTDM1	R	**0.297**	**0.451**	**0.441**
	S	*0.338*	*0.479*	*0.454*
GTDM2	R	**−0.401**	**−0.483**	**−0.485**
	S	*−0.457*	*−0.516*	*−0.498*
GTDM3	R	**−0.286**	**−0.379**	**−0.405**
	S	*−0.339*	*−0.394*	*−0.424*
GTDM4	R	**−0.301**	**−0.448**	**−0.413**
	S	*−0.372*	*−0.497*	*−0.458*
GTDM5	R	**0.250**	**0.479**	**0.423**
	S	*0.314*	*0.445*	*0.392*
FOF1	R	−0.064	**−0.220**	−0.139
	S	−0.008	−0.162	−0.083
FOF2	R	−0.020	0.156	0.076
	S	−0.051	0.159	0.079
FOF3	R	0.062	**0.249**	0.153
	S	0.011	*0.238*	0.142
FOF4	R	0.175	0.126	0.162
	S	*0.225*	*0.259*	*0.256*
FOF5	R	0.025	0.130	0.106
	S	0.119	0.124	0.124
FOF6	R	−0.053	−0.049	−0.06
	S	−0.122	−0.045	−0.084
COM1	R	0.059	0.153	0.129
	S	*0.210*	*0.211*	**0.221**
COM2	R	**−0.292**	**−0.416**	**−0.383**
	S	*−0.392*	*−0.518*	*−0.471*
COM3	R	0.174	**0.309**	**0.262**
	S	*0.303*	*0.447*	*0.384*
COM4	R	−0.175	**−0.249**	−0.192
	S	−0.156	**−0.229**	−0.152
COM5	R	**0.296**	**0.464**	**0.429**
	S	*0.353*	*0.465*	*0.429*
COM6	R	−0.064	**−0.219**	−0.139
	S	−0.009	−0.162	−0.083
COM7	R	−0.172	**−0.244**	−0.187
	S	−0.155	*−0.227*	−0.149
COM8	R	−0.105	−0.129	−0.14
	S	−0.181	−0.148	−0.168
COM9	R	**−0.24**	**−0.366**	**−0.344**
	S	*−0.324*	*−0.399*	*−0.371*
COM10	R	**−0.214**	**−0.279**	**−0.256**
	S	*−0.396*	*−0.52*	**−0.474**
COM11	R	**−0.298**	**−0.472**	**−0.433**
	S	*−0.357*	*−0.476*	*−0.433*
COM12	R	**−0.278**	**−0.467**	**−0.427**
	S	*−0.332*	*−0.472*	*−0.432*
COM13	R	0.155	**0.293**	**0.247**
	S	*0.286*	*0.461*	*0.433*
COM14	R	0.109	0.102	0.109
	S	0.110	0.087	0.109

**Table 3:** Neural networks’ confusion matrices for the testing datasets.

Class	PM_10_ AGH (MLP 17−9−3)			PM_10_ WIOS (MLP 18−9−3)			PM_2.5_ WIOS (MLP 17−12−2)	
	Good	Poor	Very Poor	Good	Poor	Very Poor	Good	Poor
good	11	0	0	10	0	0	2	2
poor	1	2	0	1	1	1	1	10
very poor	0	0	0	0	0	1	-	
Quality	92.9%			85.7%			80%	

## Data Availability

Publicly available datasets from The Voivodeship Inspectorate of Environmental Protection in Krakow were analyzed in this study. This data can be found here: (https://powietrze.gios.gov.pl/pjp/current/station_details/chart/400, accessed on 1 June 2021).
